# The hope and the hype of artificial intelligence for syncope management

**DOI:** 10.1093/ehjdh/ztaf061

**Published:** 2025-06-26

**Authors:** Samuel L Johnston, E John Barsotti, Constantinos Bakogiannis, Artur Fedorowski, Fabrizio Ricci, Eric G Heller, Robert S Sheldon, Richard Sutton, Win-Kuang Shen, Venkatesh Thiruganasambandamoorthy, Mehul Adhaduk, William H Parker, Arwa Aburizik, Corey R Haselton, Alex J Cuskey, Sangil Lee, Madeleine Johansson, Donald Macfarlane, Paari Dominic, Haruhiko Abe, B Hygriv Rao, Avinash Mudireddy, Milan Sonka, Roopinder K Sandhu, Rose Anne Kenny, Giselle M Statz, Rakesh Gopinathannair, David Benditt, Franca Dipaola, Mauro Gatti, Roberto Menè, Alessandro Giaj Levra, Dana Shiffer, Giorgio Costantino, Raffaello Furlan, Martin H Ruwald, Vassilios Vassilikos, Milena A Gebska, Brian Olshansky

**Affiliations:** Division of Cardiovascular Medicine, Division of Cardiology, Department of Internal Medicine, Roy J. and Lucille A. Carver College of Medicine, University of Iowa, 200 Hawkins Drive, Iowa City, IA 52242, USA; Department of Epidemiology, College of Public Health, University of Iowa, Iowa City, IA, USA; Department of Cardiology, Aristotle University of Thessaloniki, Thessaloniki, Greece; Department of Cardiology, Karolinska University Hospital, Stockholm, Sweden; Department of Medicine, Karolinska Institute, Stockholm, Sweden; Department of Neurosciences, Imaging and Clinical Sciences, Institute for Advanced Biomedical Technologies, University G. d’Annunzio, Chieti, Italy; Division of Cardiovascular Medicine, Division of Cardiology, Department of Internal Medicine, Roy J. and Lucille A. Carver College of Medicine, University of Iowa, 200 Hawkins Drive, Iowa City, IA 52242, USA; Libin Cardiovascular Institute, Cumming School of Medicine, University of Calgary, Calgary, Alberta, Canada; Department of Cardiology, Hammersmith Hospital, National Heart & Lung Institute, Imperial College, London, UK; Department of Cardiovascular Diseases, Mayo Clinic Arizona, Phoenix, AZ, USA; Department of Emergency Medicine and School of Epidemiology and Public Health, University of Ottawa, Ottawa, Ontario, Canada; Division of Cardiovascular Medicine, Division of Cardiology, Department of Internal Medicine, Roy J. and Lucille A. Carver College of Medicine, University of Iowa, 200 Hawkins Drive, Iowa City, IA 52242, USA; Division of Cardiovascular Medicine, Division of Cardiology, Department of Internal Medicine, Roy J. and Lucille A. Carver College of Medicine, University of Iowa, 200 Hawkins Drive, Iowa City, IA 52242, USA; Division of Hematology-Oncology and Bone Marrow Transplant, Department of Internal Medicine, Roy J. and Lucille A. Carver College of Medicine, University of Iowa, Iowa City, IA, USA; Department of Neurology, Roy J. and Lucille A. Carver College of Medicine, University of Iowa, Iowa City, IA, USA; Division of Cardiovascular Medicine, Division of Cardiology, Department of Internal Medicine, Roy J. and Lucille A. Carver College of Medicine, University of Iowa, 200 Hawkins Drive, Iowa City, IA 52242, USA; Department of Emergency Medicine, Roy J. and Lucille A. Carver College of Medicine, University of Iowa, Iowa City, IA, USA; Department of Clinical Sciences, Lund University, Malmö, Sweden; Department of Cardiology, Skåne University Hospital, Malmö, Sweden; Division of Hematology-Oncology and Bone Marrow Transplant, Department of Internal Medicine, Roy J. and Lucille A. Carver College of Medicine, University of Iowa, Iowa City, IA, USA; Division of Cardiovascular Medicine, Division of Cardiology, Department of Internal Medicine, Roy J. and Lucille A. Carver College of Medicine, University of Iowa, 200 Hawkins Drive, Iowa City, IA 52242, USA; Department of Heart Rhythm Management, University of Occupational and Environmental Health, Kitakyushu, Japan; Division of Pacing and Electrophysiology, KIMS Hospitals, Hyderabad, India; The Iowa Initiative for Artificial Intelligence, Iowa Institute for Biomedical Imaging, University of Iowa, Iowa City, IA, USA; The Iowa Initiative for Artificial Intelligence, Iowa Institute for Biomedical Imaging, University of Iowa, Iowa City, IA, USA; Libin Cardiovascular Institute, Cumming School of Medicine, University of Calgary, Calgary, Alberta, Canada; Department of Medical Gerontology, School of Medicine, Trinity College, Dublin, Ireland; Division of Cardiovascular Medicine, Division of Cardiology, Department of Internal Medicine, Roy J. and Lucille A. Carver College of Medicine, University of Iowa, 200 Hawkins Drive, Iowa City, IA 52242, USA; Kansas City Heart Rhythm Institute, Overland Park, Kansas City, KS, USA; Cardiovascular Division, University of Minnesota, Minneapolis, MN, USA; Internal Medicine, IRCCS Humanitas Research Hospital, Via Manzoni 56, Rozzano, Italy; IBM, Active Intelligence Center, Bologna, Italy; Department of Medicine and Surgery, University of Milano-Bicocca, Milan, Italy; Heart Rhythm Department, Clinique Pasteur, Toulouse, France; Department of Biomedical Sciences, Humanitas University, Via Rita Levi Montalcini 4, Pieve Emanuele, Milan, Italy; Department of Biomedical Sciences, Humanitas University, Via Rita Levi Montalcini 4, Pieve Emanuele, Milan, Italy; Emergency Department, IRCCS Ca’ Granda, Ospedale Maggiore, Milan, Italy; Internal Medicine, IRCCS Humanitas Research Hospital, Via Manzoni 56, Rozzano, Italy; Department of Biomedical Sciences, Humanitas University, Via Rita Levi Montalcini 4, Pieve Emanuele, Milan, Italy; Department of Cardiology, Copenhagen University Hospital Gentofte, Copenhagen, Denmark; Department of Cardiology, Aristotle University of Thessaloniki, Thessaloniki, Greece; Division of Cardiovascular Medicine, Division of Cardiology, Department of Internal Medicine, Roy J. and Lucille A. Carver College of Medicine, University of Iowa, 200 Hawkins Drive, Iowa City, IA 52242, USA; Division of Cardiovascular Medicine, Division of Cardiology, Department of Internal Medicine, Roy J. and Lucille A. Carver College of Medicine, University of Iowa, 200 Hawkins Drive, Iowa City, IA 52242, USA

**Keywords:** Syncope, Artificial intelligence, Hope, Hype, Clinical management, Patient experience

## Abstract

**Aims:**

Syncope remains a diagnostic challenge despite advancements in testing and treatment. Cardiac syncope is an independent predictor of mortality and can be difficult to distinguish from other causes of transient loss of consciousness (TLOC). This paper explores whether artificial intelligence (AI) can improve the evaluation and management of patients with syncope.

**Methods and results:**

We conducted a literature review and incorporated the opinions of experts in the fields of syncope and AI. The cause of TLOC is often unclear, hospitalization criteria are ambiguous, diagnostic tests are frequently non-informative, and assessments are costly. Patients are left with unanswered questions and limited guidance. Artificial intelligence (AI) has the potential to optimize syncope evaluation by processing large data sets, detecting imperceptible patterns, and assisting clinicians. However, AI has limitations, including errors, lack of human empathy, and uncertain clinical utility. Liability issues further complicate its integration. We present three viewpoints: (i) AI is crucial for advancing syncope management; (ii) AI can enhance the patient experience; and (iii) AI in syncope care is inevitable.

**Conclusion:**

Artificial intelligence may improve syncope diagnosis and management, particularly through machine learning–based test interpretation and wearable device data. However, it has yet to surpass human clinical judgment in complex decision-making. Current challenges include gaps in understanding syncope mechanisms, AI interpretability, generalizability, and clinical integration. Standardized diagnostic approaches, real-world validation, and curated data sets are essential for progress. Artificial intelligence may enhance efficiency and communication but raises concerns regarding confidentiality, bias, inequities, and legal implications.

## Introduction

**Figure 1:**
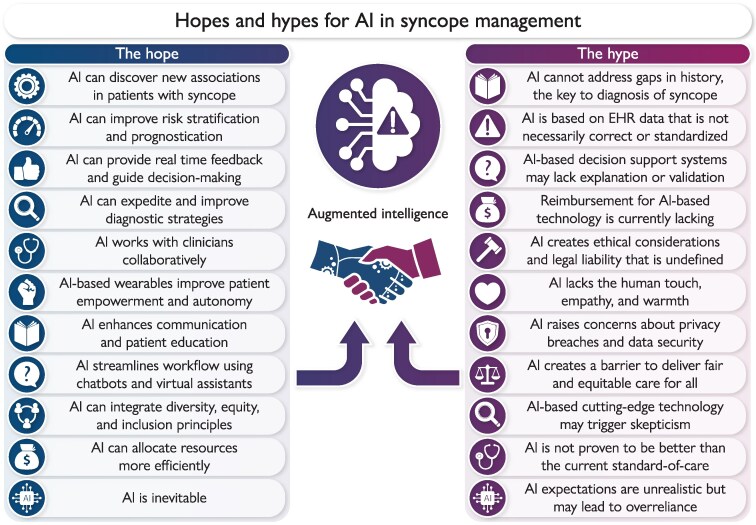
Hopes and hypes for artificial intelligence in syncope management.

Artificial intelligence (AI) is changing the practice of medicine. Artificial intelligence may extend clinicians’ diagnostic acumen and help identify the best management for those patients who experience transient loss of consciousness (TLOC).^1–5^ Here, we address the hope and hype for AI to improve management of patients with syncope by analysing three viewpoints (*Figure 1*): (i) AI is crucial to advance management of syncope; (ii) AI will improve the patient experience; and (iii) the future application of AI to syncope is inevitable.

## Artificial intelligence for clinicians

Artificial intelligence refers to computational systems that perform tasks typically attributed to human intelligence, such as learning, reasoning, problem-solving, understanding natural language, and interpreting visual data.^6–8^ Machine learning (ML), a form of AI, is characterized by automated iterative learning from a training data set.^9^ Performance is assessed on a validation or test data set. An ML algorithm can be as simple as linear regression, or as complex as a deep neural network.^10^ In either case, the principles of ML algorithms remain the same.

Deep learning applications employ neural networks that mimic the synaptic structure of the brain as computational models. Key components include (i) an input layer, consisting of units that receive data (analogous to the neuronal afferent limb); (ii) hidden layers that process data (analogous to synapses) through weighted connections and activation functions; and (iii) an output layer that generates an effect (analogous to the response/action via the neuronal efferent limb), such as a prediction or classification. Weights within hidden layers adjust to modulate the output as desired.

A neural network can have dozens of hidden layers and millions of weights, making it difficult to understand how it predicts or classifies. For example, OpenAI’s ChatGPT, with 175 billion weights, is a sophisticated generative AI large language model (LLM), with a neural network backbone called a ‘transformer’. While the principle of minimizing a loss function remains the same within all neural networks, an objective criterion for evaluating the accuracy of the model underpins much of neural network training; achieving optimal performance involves various goals and methodologies. These include heuristic-based optimization, rule-based logic, and focus on maximizing certain metrics (e.g. reward in reinforcement learning) alongside, or instead of, minimizing error.

Deep learning techniques, such as convolutional neural networks (CNNs) and long short-term memory (LSTM), have already demonstrated human-level (or beyond) interpretation of visual data including echocardiograms and electrocardiogram (ECG) tracings.^11–13^ Natural language processing (NLP) algorithms enable AI to analyse and create text and speech. However, image analysis and language analysis are discrete functions that are far less complex than the practice of medicine. Managing patients with syncope requires discernment of imperfect or incorrect data, as well as the consideration of multiple probabilities, uncertainties, and outcomes by humans.

Artificial intelligence is categorized into different levels based on its capabilities. The current state of AI, known as Narrow AI (Weak AI), is designed to perform specific tasks or solve particular problems. In contrast, General AI (Strong AI or Artificial General Intelligence) is a hypothetical advancement in which AI would possess cognitive abilities comparable with human intelligence. Beyond that, Super AI (Artificial Superintelligence) is a theoretical concept where AI would surpass human intelligence in all aspects. In modern applications, Narrow AI is sometimes referred to as *augmented intelligence*, where humans remain actively involved (*human in the loop*), and AI serves to enhance rather than replace human intelligence.

## Viewpoint 1: artificial intelligence is crucial to advance management of patients with syncope

### The current impasse in syncope management and the role of artificial intelligence

Despite decades of epidemiological studies, advances in understanding physiological processes, evidence-based risk calculators, and new diagnostic tools, challenges with identification, diagnosis, risk-stratification, hospitalization, and need for meaningful, outcome-based, interventions remain.^14,15^ The initial syncope assessment depends on a careful history, the physical examination, and an ECG.^16,17^ Nevertheless, patients hospitalized for syncope often fail to receive a definitive diagnosis and, thus, further management is uncertain. Even with expert involvement and a comprehensive workup, including cardiovascular autonomic tests, between 10 and 20% of patients remain undiagnosed, posing a possible threat for themselves, society, and care providers.^18,19^ Further, although implementation of implantable cardiac monitors meant a huge progress in unexplained cases, still, about half of monitored patients, based on ∼4400 observations, lack a final diagnosis.^20^

Artificial intelligence can identify non-intuitive, subclinical signals, find new disease associations, provide immediate feedback to clinicians, and help guide real-time decision-making through screening of the entire medical record, improve risk stratification, predict hospital length-of-stay, expedite and improve diagnostic strategies, standardize assessment, and guide management (*Figure 1*).^2,4,21^ Barriers to implementing AI while ensuring quality diagnostic and treatment guidance remain, at least in part due to lack of external validation of these models.^13,21^ Current AI models failed to surpass emergency department (ED) physicians assessing Canadian Syncope Risk Score retrospectively.^22^ Furthermore, during similar syncope risk assessment, Grant *et al.*^23^ found no statistically significant benefit from AI-based predictive models compared with traditional risk scoring methodology. However, these studies were limited by a relatively small training data set (∼4000 patients), fragmentary medical record data and the subjective nature of the predictors used.^22,23^ Recently, a syncope 30-day adverse outcomes predictive model based on ML (XGBoost model) was compared with a second model combining XGBoost predictors with knowledge-based rules, obtaining a hybrid model. The hybrid model, which relied solely on patient history, vital signs, and ECG at ED presentation, was found to outperform the single XGBoost effectiveness in predicting 30-day severe outcomes. This suggests that the synergy resulting from keeping the ‘human in the loop’, i.e. adding the human knowledge to AI-based automatic syncope risk assessment, may help to overcome the impasse.^24^

There is a need for diverse, curated syncope management data sets, potentially cloud-based, from which ML algorithms can be trained. Subsequently, additional clinical trials will be needed to compare AI-augmented management with traditional approaches for patients with syncope (*Figure 1*).

### Can artificial intelligence assist in the diagnostic evaluation?

Traditional diagnostic modalities—including ECG monitoring, tilt-table testing, active standing, and 24 h ambulatory blood pressure monitoring—are valuable for predicting or identifying potential mechanisms of syncope. However, they may lack the sensitivity and specificity needed to pinpoint the exact cause in individual patients.^25,26^ Artificial intelligence has shown promise in enhancing physicians’ efficiency and diagnostic accuracy, particularly in detecting or even predicting certain arrhythmias. It can classify rhythms during triggered events and improve the workup for arrhythmogenic syncope.^27,28^ Deep neural networks have also outperformed traditional automated methods in interpreting 12-lead ECGs, both in EDs and other clinical settings.^11^ Additionally, AI-based tools can provide timely second opinions, which may be particularly beneficial in rural or underserved areas with limited access to syncope specialists.

Interestingly, ML-based ECG interpretation has demonstrated the ability to predict both past and future atrial fibrillation (AF), offering potential utility in identifying tachy-brady syndrome–related syncope in patients with previously undiagnosed paroxysmal AF.^29^ Expanding this predictive capability to VT/VF detection could significantly improve the prevention of cardiogenic syncope and sudden cardiac death (SCD).

Implanted cardiac monitors and, more recently, wearable sensor-based technologies—such as smartwatches with photoplethysmography (PPG)—can capture sporadic events along with position and activity data, facilitating real-time prediction and diagnosis of syncope.^30,31^ Smartwatches and vest systems have also successfully assessed precipitous drops in blood pressure.^32^ Additionally, neural networks have been utilized to predict positive head-up tilt test results.^33^ However, further advances are needed to incorporate a broader range of physiologic parameters for more comprehensive analysis.

#### Wearables

Ambulatory electrocardiographic and ambulatory blood pressure monitoring provides limited and often inferential data as syncopal events are sporadic, unpredictable, and easily missed. Consequently, there is a critical need for wearable technologies capable of real-time, continuous physiological monitoring to improve diagnostic accuracy by recording actual syncopal events or physiological alterations that may be highly predictive of a pathophysiological aetiology of syncope. The widespread use and accessibility of over-the-counter wearable sensor-based technologies (wearables), such as smartwatches, present opportunities to improve diagnostic acumen. The Internet of Things (IoT) enables wearables to instantly alert emergency medical services or a physician’s office to critical health findings. This connectivity could also facilitate the transmission of anonymized population data to cloud-based servers, where AI analysis of big data could be used to better understand what constitutes normal physiology and what is pathophysiological.

The ability of wearables to capture events as they occur and provide key physiological signals extends beyond heart rate and rhythm. These include such parameters as heart rate variability (a proxy for autonomic tone), blood pressure (and its temporal relationship to heart rate changes to capture autonomic changes), tissue oxygenation, activity, position/posture, muscle tonicity (e.g. intentional movements, tonic-clonic muscle activity during seizure and flaccidity with syncope), and even electroencephalography. Wearables can help correlate symptoms to events to help capture causality [e.g. ventricular ectopy (VE) in relation to symptoms]. While no single wearable solution currently meets all diagnostic needs, ML-driven multimodal integration of these signals may yield a more comprehensive assessment of syncope aetiologies beyond present diagnostic capabilities.

Therefore, the role of ML extends beyond data collection, signal processing, signal cleaning, and interpretation. New physiologic correlates can emerge via annotation of complex physiological data and integration of multimodal signals to contextualize syncope events. Recent AI-driven analyses of ambulatory electrocardiographic data already show superiority over human analyses, highlighting the need for further validation in wearable-derived data sets.^34^ Additionally, ML techniques such as periodic and event-based analyses could enable deeper insights into physiological patterns, distinguishing diagnostic from prognostic indicators of syncope and better defining what is normal.

Currently, PPG is the predominant technology used in consumer wearables. However, it has several limitations. It is prone to motion artefacts and ambient light leakage. For heart rate and rhythm assessment, PPG can only infer rhythm regularity, using irregularity as a proxy for AF. Also, it is inherently unable to detect VE, highlighting the need for improved PPG algorithms or the integration of more sensitive and specific wearable detection methods for VE.

Photoplethysmography also has significant limitations in blood pressure estimation. First, it measures volumetric changes in peripheral circulation, typically at the fingers or wrist. Second, its accuracy depends heavily on the vascular condition at the measurement site, making it prone to errors in individuals with peripheral vascular disease. Third, abnormal vasoconstriction can introduce further inaccuracies. Lastly, the sensor’s position relative to heart level is crucial, and without proper correction—often unavailable in commercial devices—readings may be unreliable.^35^

While wearable technology has already reshaped syncope diagnostic capabilities, the integration with ML will refine and accelerate its clinical impact. The ability to process and interpret diverse physiological signals in real time will improve diagnostic accuracy, personalize patient management, and ultimately improve outcomes. Moving forward, a concerted effort is needed to enhance wearable sensors, refine ML-driven analytics, and validate these innovations in large-scale clinical studies.

#### Natural language processing

Beyond ECG interpretation and wearables, AI-driven NLP techniques are being explored to improve diagnostic assessments. ML methods, such as LSTM networks, can analyse patient narratives, helping differentiate syncope from other TLOC events. Supervised learning techniques and time series analysis are particularly well suited for evaluating large electronic health record (EHR) data sets, enabling more accurate classification of syncope causes and guiding therapy selection. Beyond chart review, NLP also has the potential to assist in guiding patient history taking.

A recent study examined syncope outcomes in ED patients, predicting hospitalization, ICU admission, and in-hospital mortality.^36^ Notably, ML models outperformed unaided physician classification in predicting ICU admissions and mortality. Future research may further refine syncope classification (e.g. cardiac vs. non-cardiac) and develop AI-driven therapeutic recommendations (see Can artificial intelligence assist physicians in the treatment of patients with syncope?).

### Can artificial intelligence assist physicians in the treatment of patients with syncope?

Before planning any intervention, it is prudent to consider a personalized approach to treatment that prioritizes the simplest, least risky recommendations, including patient education, reassurance, adequate fluid intake, counter-pressure manoeuvres, and avoidance of triggers to prevent recurrence in patients with reflex syncope. For patients with reflex syncope, a clear and detailed explanation of why they passed out has value that AI could provide. Natural language processing–driven patient communication systems can help clinicians bridge the educational gap (see Enhanced education, communication, and support after a syncopal event), improve patient quality of life, and prevent recurrent syncope.^37^

If conservative measures fail, treatment plans depend on the aetiology of syncope. Cluster analysis, a form of unsupervised learning, could assist in formulating empirical treatment plans by identifying patterns in patient phenotypes that correlate with successful outcomes from specific treatment regimens, even in the absence of a definitive diagnosis. This approach can generate insights regarding treatment of syncope based on similarity to other patients rather than by establishing causality. The effectiveness of this strategy depends on careful application and interpretation within the broader context of clinical research and knowledge. It will require sufficiently large sample sizes of successfully managed patients with similar conditions to generate benefit.

If pharmacologic intervention is needed, EHRs are equipped with practice advisory warnings on QT interval prolongation, drug–drug interactions, and allergies. Artificial intelligence can further enhance this feature by uncovering new interactions between medications and poor outcomes.^38^

Artificial intelligence can guide individualized therapy and assist with use of practice guidelines, evidence-based international registries (perhaps, created by AI and cloud-based), and other data. Likewise, evidence collected by AI may provide insight into which patients (if any) with cardioinhibitory and carotid sinus syncope could benefit from pacemaker implantation or cardioneuroablation despite lack of controlled clinical trials.

While AI-assisted syncope management shows promise, its clinical adoption is limited by a lack of robust validation. There is a shortage of well-designed studies assessing its safety, efficacy, and clinical utility. Furthermore, AI models require large, well-curated data sets with standardized diagnostic criteria to improve accuracy and reliability. Despite these challenges, emerging AI technologies—such as automated ECG analysis and EHR-based risk stratification—offer hope for enhancing syncope diagnosis and management.

The following hypothetical cases illustrate the potential application of AI to the management of patients with syncope (Appendix).

**Appendix:**
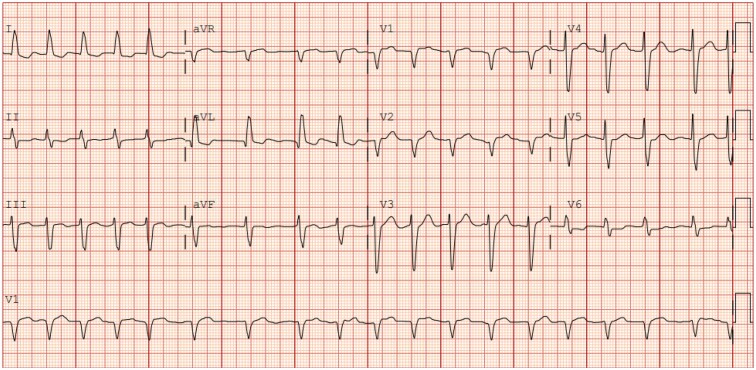
Comparison of traditional and artificial intelligence–based approaches to evaluating patients with syncope (hypothetical cases).

## Viewpoint 2: artificial intelligence will improve patient experience

Artificial intelligence has the potential to expedite care, improve doctor–patient communication, provide patients with an understanding of their disease, suggest medications, and recommend personalized therapy.

### Effective workflow to streamline syncope patients

At the institutional level, AI-assisted systems have the potential to improve patient experience through mitigating ineffective workflows and shortening wait times.^39^ These efficiencies will be augmented by the implementation of AI-driven pre-consultation history collection and needs assessments for laboratory and imaging services (*Figure 1*).^39,40^ The median wait time in EDs is recently 6.58 h at 85% capacity, and patients with syncope may wait even longer. The percentage of patients leaving the ED without being evaluated has surged to 10%.^41^ In the context of syncope-related ED visits, AI could optimize resource utilization towards high-risk patients and away from those safe to discharge. In the ambulatory setting, AI-based ECG signal processing has been shown to reduce clinician workload by 42% in patients being monitored by implantable cardiac monitors.^27^ These data support AI’s potential to expedite syncope care and broaden access to healthcare.

### Enhanced education, communication, and support after a syncopal event

Artificial intelligence can improve patient experience by enhancing communication and overcoming cultural and linguistic barriers. It can assist clinicians by facilitating and augmenting patient education and addressing inquiries.^37,42,43^ Access to AI-based solutions, including LLM chatbots derived from published data and clinical guidelines, has the potential to reliably answer the syncope patient’s questions, as well as to help them prepare questions for their clinicians. It can strengthen patient engagement and alleviate anxiety through various behavioural techniques using smartphone applications.^44^ It can also help guide clinician responses to patient concerns and facilitate timely communication.

Although AI-based algorithms have significantly advanced in recent years, rigorous validation and testing remain crucial to ensuring reliable ML outcomes and maintaining trust. Artificial intelligence can produce erroneous information, including ‘hallucinations’—plausible but fictitious results.^45^ Another notable limitation of LLMs, such as ChatGPT, is their inability to consistently provide accurate medical references.^46^ For these reasons, human oversight remains essential.

While these pitfalls are likely to be overcome and the incorporation of AI-powered technology to personalize patient education using credible resources may be realized, the lack of human interactions (facial expressions, gestures, touch, and other non-verbal cues) can still create scepticism.^47^ Therefore, AI algorithms will need to account for a diverse range of individual preferences, values, and emotional responses that impact healthcare decisions and reflect the complexity of human emotions.

### Artificial intelligence and the perception of empathy in syncope management

While genuine human empathy is often considered a cornerstone of effective patient care, it is ultimately the patient’s perception of empathy that matters. Patients do not require their clinicians—or the AI systems assisting them—to experience emotions in order to feel that they are heard, understood, and supported. In fact, evidence suggests that AI may outperform human clinicians in conveying what patients interpret as empathy. A 2023 study in *JAMA Internal Medicine* found that patients preferred chatbot-generated responses to those from physicians nearly 80% of the time.^48^ A 2024 study in *PNAS* similarly demonstrated that chatbots were perceived as more empathetic than human responders, largely because they avoided common conversational pitfalls.^49^ Unlike humans, AI does not redirect conversations to personal experiences, rush to offer solutions, or allow fatigue and time constraints to affect the quality of interactions. Instead, it consistently acknowledges patient concerns, validates emotions, and maintains focus on the patient’s experience.

For patients with syncope, this ability may be especially relevant. The uncertainty surrounding a syncopal episode, combined with prolonged wait times and inconsistent communication, can heighten anxiety. Artificial intelligence–driven systems can provide immediate, structured, and emotionally intelligent responses, reinforcing patient confidence and engagement. While AI does not possess human emotions, it can reflect the needs of the patient in a personalized manner without imbuing its own identify, making it a potentially more reliable source of perceived support than an overburdened clinician.

However, the long-term effectiveness of AI in sustaining perceived empathy remains uncertain. While chatbots may excel in isolated interactions, their structured approach could feel repetitive or impersonal over time. Additionally, some patients may remain sceptical of AI-driven care, preferring human interaction despite evidence suggesting that chatbot communication is often perceived as more compassionate. A trusting relationship between the clinician and the patient should be preserved. Healthy boundaries between anthropomorphized LLMs and patients will need to be established. As AI technology continues to evolve, its role in patient experience will likely be as an adjunct rather than a replacement, reinforcing empathetic communication where human limitations exist.

## Viewpoint 3: artificial intelligence is inevitable

Artificial intelligence systems can perform functions impossible for humans. Neural networks can detect subtle patterns on ECGs imperceptible to the human eye and even predict future arrhythmias^29,50^ (see Can artificial intelligence assist in the diagnostic evaluation?). Artificial intelligence can already accurately predict conditions that cause syncope, including concealed long QT syndrome, Brugada syndrome, hypertrophic cardiomyopathy, aortic stenosis, and ventricular dysfunction.^51–53^ Artificial intelligence algorithms could potentially predict syncope before it occurs, diagnose the root cause after an event, and recommend life-saving treatment. While these possibilities suggest an inevitable role for AI in syncope management, questions remain (*Table 1*).

**Table 1:** Comparison of physician-based vs. artificial intelligence–driven syncope management

Aspect	Physician-based approach	AI-driven approach
Diagnosis	Mostly relies on patient history, physical exam, ECG, and event recorders. Subjective interpretation of symptoms and based on experience diagnosis	AI-enhanced models for ECG, BP, and continuous sensor-based monitoring. Easier to detect patterns missed by humans, while also mining information and data that might be helpful
Risk stratification	Mainly based on experience, intuition and clinical judgment, scoring systems (e.g. San Francisco Syncope Rule), and cardiac telemetry monitoring	AI models integrate real-time patient data from wearables and IoT devices, prior cases, and predictive analytics for risk assessment
Monitoring and follow-up	Intermittent monitoring with Holter monitors or ILRs; follow-ups based on fixed schedules and the availability of resources	Continuous real-time monitoring with wearables and IoTs, AI-powered ILRs, and remote patient monitoring platforms
Personalized care	Treatment decisions based on physician experience and general guidelines, leading to more one size-fits-all treatment plans and solutions	AI-driven personalized treatment plans based on individual patient profiles via EHR and other data sources and real-time data monitoring
Sources and use of data	Limited to fragmented sources of data including patient history, ECG, and occasional telemetry; retrospective analysis of data and based on prior experience and guidelines	Big data analysis from multiple sources and AI-enhanced driven data mining and data analysis (wearables, electronic health records, AI analytics)
Error and bias rates	Potential for human errors due to cognitive bias, fatigue, or misinterpretation	Reduces human error but risks algorithmic bias; requires robust validation frameworks for continuous evaluation of AI-enhanced tools accuracy and safety
Interpretability and transparency	Physicians explain findings to patients, but interpretation varies by expertise and experience	AI models and LLMs lack transparency (‘black box’ and hallucinations). Explainable AI (XAI) aims to improve interpretability. Medical-focused LLMs are under development for education and medical information interpretation
Efficiency	Limited by physician and resources availability. Delays in diagnosis, treatment, and follow-up could be observed	Real-time data processing from sources such as wearables and IoTs allow instant alerts for high-risk events. Faster intervention
Cost and environmental implications	High costs for repeated physician visits, diagnostic tests, and hospitalizations	Potential to reduce costs by optimizing diagnostics and reducing unnecessary hospital admissions. Cost-effectiveness and cost-benefit analysis is needed. The environmental challenges (electricity, water for cooling, etc.) from a broader implementation of AI tools should be taken into consideration to develop mitigation strategies
Human factors and patient experience	Patient interaction allows for empathy and reassurance but may vary between physicians and time constraints	Limited direct human interaction. Optimized hybrid models to maintain patient trust and engagement might be preferred

Concerns surrounding privacy, bias, and trust may prevent or delay AI systems from being incorporated into syncope management (*Table 1*). Artificial intelligence models require numerous examples to develop pattern recognition. Useful data sets include personal health information from large numbers of patients. Even if big data can improve healthcare, de-identification of patient health information remains a problem. Artificial intelligence algorithms sometimes contain intrinsic systemic biases augmenting disparities. Racial and ethnic biases, among others, in some AI algorithms have been documented.^54^ The usefulness of AI is subject to the generalizability of the data used to train it. *Data drift*, where the data set used to train a ML algorithm no longer represents the data encountered during deployment, is a significant concern.^55^ Contemporary EHR systems contribute to lack of heterogeneous data sets. Poor representation of some populations in AI syncope databases will lead to biased treatment benefits and inequity, potentially resulting in inaccurate predictions and concerns about management.

Alternatively, the power of AI to acquire and interpret medical knowledge independent of clinicians can improve accuracy and reduce costs. Artificial intelligence can facilitate delivery of high-quality, advanced tools to hospitals who lack sonographers, cardiologists, and radiologists. Hospital costs associated the inpatient evaluation of patients with syncope exceed $2.4 billion per year in the USA.^56^ By a variety of mechanisms, AI has the power to reduce these costs. Hospitals and clinics throughout most of the word already possess the hardware and infrastructure to incorporate AI-facilitated medicine, namely, a personal computer and an internet connection.

Already, over 60% of clinicians and trainees view AI positively.^57^ This supports its eventual incorporation into syncope management. However, widespread adoption of AI in healthcare has yet to occur, and clinicians tend to mistrust inexplicable black box models, as well as applications that are not user friendly.^58^ When first introduced, computer-based EHRs promised to improve physician efficiency. Arguably, they have had the opposite effect. Electronic health record–induced fatigue has, in fact, lowered physician efficiency.^49^ The application of AI to syncope management should be timesaving rather than time-consuming. If it is error-prone or requires exorbitant human oversight, then its incorporation into syncope management is less certain.

Barriers to implementation of AI in syncope management include ethical and legal considerations, among others (*Figure 1*). Obtaining informed consent for AI-based care and clinical research may prove challenging.^54,59^ Guidelines ensuring transparency in data collection and algorithmic decision-making, grounded in a gold standard or definitive reference for syncope diagnosis, are needed. Integration of AI into the management of syncope requires that concerns of privacy, liability, trust, and bias are addressed thoughtfully.

## Conclusions

Syncope remains a challenging clinical problem, with causes often obscure, unclear criteria for hospitalization, and diagnostic tests that frequently yield non-diagnostic results, leaving patients uncertain about their diagnosis and management. While AI shows promise in improving syncope management by enhancing risk stratification, streamlining diagnostic pathways, and optimizing resource utilization, it has not yet surpassed human capabilities, particularly in complex clinical judgment. Current applications are limited by gaps in understanding syncope mechanisms, challenges with AI interpretability, generalizability, and clinical integration. Rigorous validation in real-world settings, alongside standardization of diagnostic approaches using curated data sets, is essential. Clinical trials will be critical to demonstrate that AI-augmented syncope management improves outcomes. Artificial intelligence also offers potential benefits, including improved patient experiences through enhanced efficiencies and better communication, but concerns regarding confidentiality, biases, inequities, and legal implications remain. Based on our present state of knowledge, and in the context of narrow AI, it is imperative for clinicians to maintain their clinical skills and a central role in decision-making. This ensures that AI serves as an adjunct to, rather than a replacement for, clinical judgment, allowing clinicians to recognize when AI may be in error.

## Data Availability

No new data were generated or analyzed in support of this manuscript. As this is a review and perspective piece, all data referenced are from previously published sources, which are cited appropriately in the text.

## Lead author biography

Dr. Johnston is a clinical cardiac electrophysiologist who serves as a Clinical Associate Professor of Medicine at the University of Iowa Hospitals & Clinics and interim Deputy Chief of Cardiology at the Iowa City Veterans Affairs Medical Center. He completed his training in internal medicine, general cardiology, and cardiac electrophysiology at Loyola University Medical Center in Maywood, Illinois. His research interests include syncope, sudden cardiac death, PVC-mediated cardiomyopathy, and the application of artificial intelligence to clinical cardiology.

## Appendix. Comparison of traditional and AI-based approaches to evaluating patients with syncope (hypothetical cases)

**Case 1:** A 72-year-old female with permanent atrial fibrillation, Parkinson’s disease, severe orthostatic hypotension, and mild dementia presents with recurrent syncope for evaluation.

- The patient has experienced several witnessed episodes of dizziness following sudden postural changes. She reports occasional fainting episodes while sitting and watching television.
- *Vital signs*: Supine BP 137/83 mmHg, HR 97 bpm; standing BP 112/78 mmHg, HR 94 bpm.
- *Twelve-lead ECG*:
- *Echocardiography and cardiac catheterization* within the past 2 years revealed no significant structural heart disease or occlusive coronary artery disease.

*Traditional approach*: In the absence of structural heart disease, a junior physician diagnoses the patient with syncope due to orthostatic hypotension. Compression stockings are prescribed, and increased hydration is recommended.

*Artificial intelligence–enhanced approach*: The AI model expands the differential diagnosis by recognizing the patient’s risk for bradyarrhythmias due to the presence of AF and LBBB. It recommends ambulatory ECG monitoring, which promptly detects intermittent complete heart block that correlates with the patient’s syncopal episodes.

**Case 2:** A 65-year-old male with a history of hypertension and type 2 diabetes presents with an unexplained fainting episode. The patient experienced sudden syncope while shopping. On examination, no significant physical findings were noted.

- *Vital signs*: BP 130/80 mmHg, HR 78 bpm, SpO_2_ 97%; orthostatics negative.
- *Electrocardiogram*: Normal sinus rhythm, normal axis, QTc prolongation (480 ms).
- *Laboratory findings, including troponin, were within normal limits*.
- *Differential diagnosis*: Benign reflex syncope vs. cardiac or arrhythmia-related syncope.

*Traditional approach*: Observation in the ED for several hours, with a suggestion for outpatient Holter monitoring. Based on the ESC syncope guidelines, the patient is classified as low-to-intermediate risk.

*Artificial intelligence–enhanced approach*: The AI risk stratification model integrates historical EHR data, real-time ECG from wearables/IoT devices, and syncope presentation patterns from similar cases in the cloud to refine risk assessment. The AI model flags the patient as high risk based on personalized risk profiling. Holter monitoring reveals intermittent polymorphic ventricular tachycardia (VT), and early intervention with an implantable cardioverter-defibrillator (ICD) is recommended.

**Case 3:** A 15-year-old male presents with two episodes of syncope during football training. He has no prior history of syncope or medical conditions.

- *Electrocardiogram*: 12-lead ECG shows borderline sinus bradycardia with a normal QT/QTc interval.
- *Echocardiogram*: Normal findings.
- *Exercise stress test*: Equivocal for adequate QTc shortening during exercise and equivocal QTc lengthening during the first minute of recovery.
- *Genetic testing*: Reveals a KCNQ1 gene mutation, confirming long QT syndrome (LQTS) type 1.

*Traditional approach*: The physician recommends an implantable cardioverter-defibrillator (ICD) and advises avoidance of competitive sports.

*Artificial intelligence–enhanced approach*: AI-assisted wearable single-lead ECG and PPG detect early signs of LQTS type 1, including incidental QT prolongation during exercise and short runs of polymorphic non-sustained ventricular tachycardia (NSVT), before the first syncopal episode. Artificial intelligence–enhanced 12-lead ECG detects the LQTS type 1 phenotype during the pre-participation physical exam. With the aid of augmented intelligence, the physician recommends treatment with nadolol and ongoing monitoring. Artificial intelligence facilitates shared decision-making between the patient, his parents, and the physician, allowing the patient to safely choose to continue participating in athletics.

**Case 4:** A 78-year-old female was hospitalized for atypical chest pain. Her past medical history was unremarkable, as was her initial ECG. Cardiac catheterization performed due to an abnormal stress test showed a tight lesion in the first obtuse marginal that was stented successfully. Thirty minutes later, she developed nausea followed by TLOC while being transported to the recovery room. She was not monitored by telemetry during this event. Cardiac telemetry was promptly started and revealed sinus rhythm with sinus arrhythmia.

*Traditional approach*: The physician recommends overnight hospitalization with cardiac telemetry and discharges the patient to home the following day with a 48-h Holter monitor.

*Artificial intelligence–enhanced approach*: AI-assisted EHR review scans the patient’s records from other hospitals and identifies documentation of a prior syncopal episode during phlebotomy. Artificial intelligence–assisted interpretation of the patient’s telemetry immediately following her syncopal episode identifies subtle gradual lengthening of the P-P intervals, R-R intervals, and the PR lengthening of PR intervals during spasms of nausea. The patient is correctly diagnosed with vasovagal syncope. Artificial intelligence–assisted management suggests checking orthostatic vital signs and hydration status prior to hospital discharge. Upon resolution of the trigger (nausea), she was discharged to home later that day.
